# Depletion of Cell Adhesion Molecule L1 from Microglia and Macrophages Reduces Recovery After Spinal Cord Injury

**DOI:** 10.3390/ijms26073285

**Published:** 2025-04-01

**Authors:** Thomas Theis, Suneel Kumar, Pratiksha Shah, Mukti Patel, Iman Tadmori, Carlos Ayala, Monica Tschang, Wise Young, Melitta Schachner

**Affiliations:** 1 Keck Center for Collaborative Neuroscience, Department of Cell Biology and Neuroscience, Rutgers University, Piscataway, NJ 08554, USA; theisneuro@gmail.com (T.T.); pratikshashah98@gmail.com (P.S.); patel.mukti@outlook.com (M.P.); itadmori@dls.rutgers.edu (I.T.); drayala.carlos@gmail.com (C.A.); mat361@scarletmail.rutgers.edu (M.T.); wisey@dls.rutgers.edu (W.Y.); 2 Department of Biomedical Engineering, Rutgers, The State University of New Jersey, Piscataway, NJ 08844, USA; sk1350@soe.rutgers.edu

**Keywords:** L1CAM, microglia, spinal cord injury, functional recovery, Basso mouse scale

## Abstract

The young mammalian central nervous system regenerates after spinal cord injury and recovers locomotion, whereas adult mice only show limited recovery that depends on the injury severity, genetic background, and physical therapy. At the molecular level, key regulators that contribute to recovery are cell adhesion molecules, such as L1CAM (L1). At the cell surface, L1 functions as a homotypic receptor that signal-transduces crucial functions in neuronal migration and survival, neurite outgrowth, myelination, formation of synapses, and synaptic plasticity. In the adult central nervous system, L1 is expressed only by neurons. We now show that L1 is unexpectedly also expressed by 26% microglia, freshly isolated from a 7-day-old mouse brain. At postnatal day 21, only 3% of microglia are L1-positive. Using a mouse mutant in which L1 is deleted specifically in monocytes of 10- to 14-week-old mice, functional recovery was reduced up to 4 weeks after injury at lower thoracic spinal levels. Also, NF200-immunoreactive and 5-HT-immunoreactive fibers were found decreased below the injury site as compared to wild-type mice. In conclusion, microglial cells that express L1 stimulate neurite outgrowth in vitro, improve functional recovery after spinal cord injury in adult mice, and increase fiber densities caudal to the lesion site.

## 1. Introduction

Spinal cord injury (SCI) is a devastating disease with an annual worldwide incidence of about 40 cases per one million people. It affects both young and aged people, resulting in the permanent impairment or loss of voluntary motor function and sensation below the level of injury [1]. SCI has a tremendous impact on the lives of its victims and their families, with considerable financial, emotional, and psychological burdens [2]. Although there are promising results from clinical trials studying new treatments via cell implantation that are accompanied by extensive rehabilitation and epidural stimulation, these approaches only help a subset of the patients, and the molecular mechanisms underlying these benefits are not well understood [3,4].

At the molecular level, cell adhesion molecules are key regulators that enhance neuroplasticity and regeneration after neurotrauma. Cell adhesion molecules such as L1 have their peak expression levels at early postnatal days and function as signal transducers involved in cell functions. The cell adhesion molecule L1 (L1CAM, also known as CD171) is a member of the immunoglobulin superfamily of adhesion molecules [5]. From an epidemiological point of view, L1 has a prevalence of approximately 1/30,000 and is the globally predominant cause of hydrocephalus in humans. Because the L1 gene localizes to the X-chromosome, males are mainly affected. The abnormalities of the L1 syndrome depend on genetic background. Heterozygous females often do not show overt abnormalities, but these again depend on the genetic background and have not been fully investigated in human females. Many neuronal functions were shown to depend on L1, such as neuronal migration and survival, axonal outgrowth, guidance and fasciculation, synapse formation, and synaptic plasticity [5]. These functions are triggered by homophilic and heterophilic interactions with molecules of the extracellular matrix or adhesion molecules at the cell surface [5]. In addition to the essential role of L1 in the development of the central and peripheral nervous systems, it has been reported to enhance regeneration and improve functional recovery after SCI and peripheral nerve injury [6,7,8,9,10]. In addition, an antagonistic antibody of L1 increases the infarct size in a cerebral ischemia–reperfusion rat model [5]. Overexpressing L1 in the spinal cord using an adeno-associated virus led to enhanced remyelination and axonal regrowth/sprouting/sparing and improved functional recovery after SCI [5]. In addition, small organic compounds that structurally and functionally mimic L1 and act as L1 agonistic mimetics [10]. These L1 mimetic agonists improved functional recovery after SCI in mice and zebrafish [5,10,11]. The abnormalities depend on the genetic background. Heterozygous females often do not show overt abnormalities, but these again depend on the genetic background and have not been fully investigated in human females.

In an AD mouse model in which neurons overexpress mutated forms of amyloid precursor protein and presenilin-1, viral introduction of L1 into the hippocampus and occipital cortex was observed to reduce AD pathology. Importantly, L1 directly binds to amyloid-β (Aβ), but not to amyloid precursor protein, and reduces the aggregation of Aβ42 in vitro [12]. Interestingly, in the cerebrospinal fluid of AD patients, elevated levels of L1 fragments were found [12]. In a parabiosis model, the circulatory system of an 18-month-old AD mouse was joined with a healthy 2-month-old wild-type mouse, and increased levels of the 70 kDa L1 cleavage product were found in the AD hippocampus. Interestingly, these mice showed reduced amyloid plaque deposition and more activated microglia [13].

L1 is also detectable in non-neural tissues, such as skin, kidney, and sperm [14,15]. Noteworthy for the present study is that L1 is expressed by different types of leukocytes, and it plays an important role in interactions between leukocytes and endothelial cells. Remarkably, L1 is overexpressed in many malignant tissues and enhances tumor cell migration, which correlates with poor clinical outcomes [16,17,18,19,20]. Until now, L1 was described to be only expressed by neurons in the central nervous system, and enteric neurons and Schwann cells in the peripheral nervous system. It was believed that microglia, which function as central nervous system immune cells, particularly after neurotrauma, would derive from the periphery and infiltrate the brain and spinal cord [21]. However, we now describe a subpopulation of central nervous system microglial cells and infiltrating macrophages that express L1. We showed that an L1 antagonistic antibody reduces cell migration in vitro and that co-culturing of L1-positive microglia with cerebellar granule cells enhances neurite outgrowth. Specific depletion of L1 from microglial cells and macrophages reduces functional recovery, sprouting, and possibly also saving nerve fibers after SCI.

## 2. Results

### 2.1. Subpopulations of Microglial Cells Express L1

**Figure 1:**
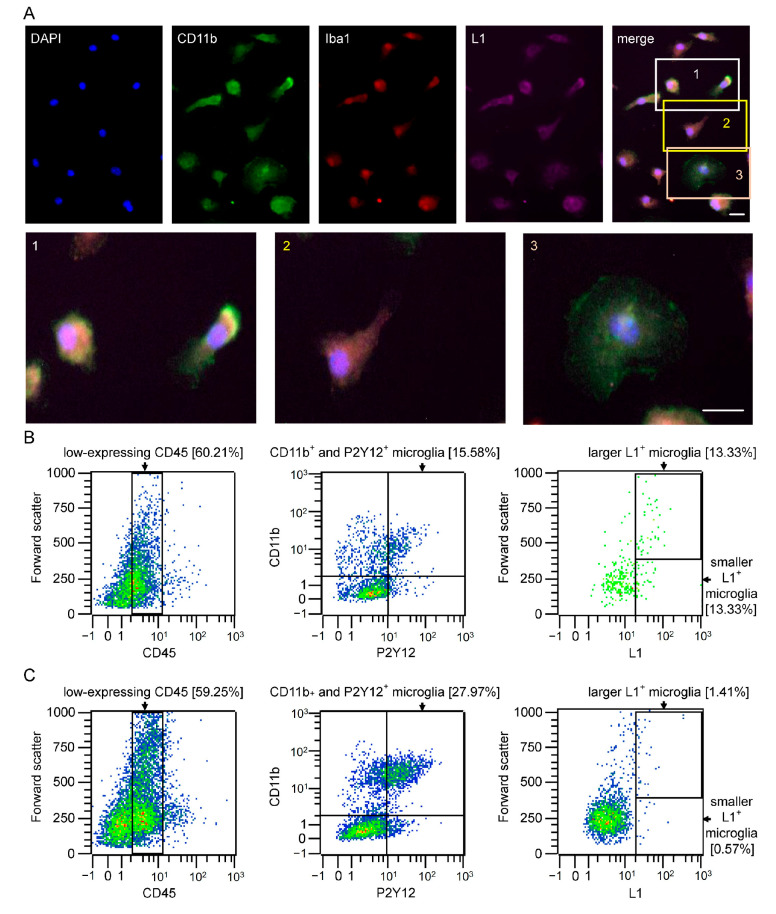
A subpopulation of microglia expresses L1: (**A**) Representative images of cultured microglial cells stained for the microglia markers CD11b (green) and Iba1 (red), for L1 (violet) and cell nuclei (DAPI, blue). The merged images show a subpopulation of microglia expressing L1. The white box numbered with 1 indicates the enlarged area with cells that highly express L1, the yellow box numbered with 2 indicates the enlarged area with cells that moderately express L1, and the orange box numbered with 3 indicates the enlarged area with cells that do not express L1. Scale bars = 30 µm. (**B**,**C**) Flow cytometry of freshly isolated microglia from pooled brains of 7-day-old mice of both sexes (*n* = 1, (**B**)) and 21-day-old mice of both sexes (*n* = 1, (**C**)). The gates were set to identify the microglia population. All microglia show lower expression of CD45 (left scatter plots) and higher expression of CD11b. P2Y12 is only expressed by microglia and not by infiltrating macrophages (middle scatter plots). The scatter plots in the panel on the right side show the percentage of microglial cells that express L1. Color gradients in the scatter plots are representing the density of cells in different regions of the plot. Blue dots indicate a low cell number, green dots indicate a moderate cell number, and red dots indicate a high cell number.

A subpopulation of microglial cells prepared from the brains of CB6F1/J mice could be shown to express L1 by immunofluorescence staining (Figure 1A). These cells were found to express 11b (CD11b) for monocytes (green), ionized calcium-binding adaptor molecule 1 (Iba1) (red) for microglial cells and macrophages, L1 (violet), and the cell nuclei (DAPI, blue). The merged images represent the co-expression of all markers within the subset of the microglia population expressing L1. The enlarged images show individual cells with different morphologies and colocalization of L1 with different microglial markers. To check for non-specific background labeling, cultured microglial cells were stained only with the corresponding secondary antibody in the absence of the primary antibody. Only minimal fluorescence was observed (Figure S1).

Freshly isolated microglia samples from 7-day-old (Figure 1B) and 21-day-old (Figure 1C) mice were prepared for flow cytometry analysis to quantify the population that expresses L1. CD45, CD11b, purinergic receptor P2Y, G-protein coupled, and 12 protein (P2Y12) were used as markers for monocytes, among which P2Y12 is specifically expressed by microglia [22]. Microglia normally express low levels of the hematopoietic stem cell marker CD45, whereas infiltrating macrophages express higher levels of CD45 [21]. Activated microglia express low levels of P2Y12. CD11b is a marker for myeloid lineage cells expressed by both microglia and macrophages. With regard to L1, approximately 27% of microglia express L1 in microglial cells from 7-day-old mice, with approximately 50% displaying a larger cell soma and the other 50% showing a smaller soma (Figure 1B). Only approximately 2% of microglial cells express L1 in 21-day-old mice (Figure 1C).

Since the L1 gene is localized on the X-chromosome, we investigated whether there are sex differences in the L1-positive microglia population. There were no differences (male = 46.49%; female = 48.9%) in 4-day-old mice (Figure S2).

### 2.2. Reducing L1 Function Inhibits Microglial Cell Migration

**Figure 2:**
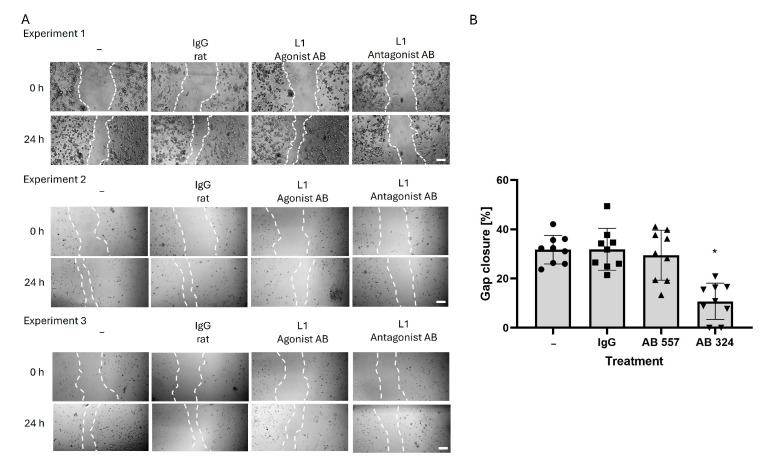
Antagonistic L1 antibody reduces microglial cell migration: (**A**) Representative images of confluent microglial cells from three experiments at 0 h and 24 h after scratch injury. Dotted lines mark the edges of the scratch. Scale bar = 100 µm. (**B**) The bar diagram shows the % average gap closure (*n* = 6 from three independent experiments, error bars indicate SD) and individual data points from all experimental groups (circle: untreated (-), square: IgG treated (IgG), triangle: L1 function-triggering antibody treated (AB 557), inverted triangle: L1 function blocking antibody treated (AB 324)). Data were analyzed using one-way ANOVA followed by post hoc Fisher’s protected least significant difference test. * *p* < 0.05.

L1 changes microglial cell migration in the scratch assay (Figure 2). The monolayer of microglial cells was subjected to a scratch and the percentage of gap closure over 24 h was measured. The untreated control group depicts the baseline of microglial cell migration. The non-immune IgG-treated control group showed a 30% gap closure that is similar to the non-treated cells. Since the gap is not fully closed under these conditions, it is unlikely that the system is saturated. The L1 agonistic monoclonal antibody did not affect migration of microglial cells. In contrast, the L1 antagonistic antibody decreased the gap closure percentage to approximately 20%, indicating that microglial cell migration is inhibited by blocking L1 function (Figure 2).

### 2.3. L1-Expressing Microglial Cells Enhance Neurite Outgrowth of Cerebellar Granule Cells

**Figure 3:**
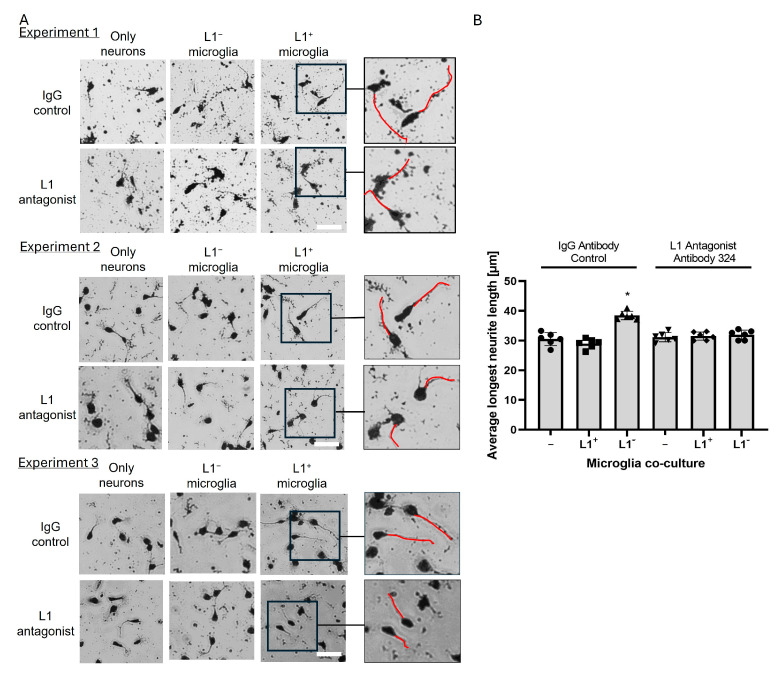
(**A**) Representative images from three experiments of cerebellar granule cells cultured either alone (Only neurons) or co-cultured with L1-negative (L1^−^ )or L1-positive (L1) microglia. Scale bars = 30 µm. Black boxes indicate the areas that were enlarged. The red lines in the enlarged images show examples of neurites. (**B**) Control L1-positive microglial cells enhance neurite outgrowth of co-cultured cerebellar granule cells. The bar diagram shows the average longest neurite length of cerebellar granule cells in co-cultures (*n* = 6 from three independent experiments) and individual data points from all experimental groups (circle: no microglia co-culture, i.e., only neurons, and treated with IgG (-), square: L1^+^ microglia co-culture and treated with IgG, triangle: L1^−^ microglia co-culture and treated with IgG, inverted triangle: no microglia co-culture and treated with L1 function blocking antibody 324, diamond: L1^+^ microglia co-culture and treated with L1 function blocking antibody 324, circle: L1^−^ microglia co-culture and treated with L1 function blocking antibody 324). Data were analyzed using one-way ANOVA followed by post hoc Fisher’s protected least significant difference test and shown as mean ± SD. * *p* <0.05.

Homophilic L1 interactions trigger neurite outgrowth. Thus, we analyzed whether microglial-expressed L1 stimulates neurite outgrowth from L1-expressing neurons. For this experiment, microglial cells were harvested from 0-day-old brains and FACS separated into L1-negative, i.e., control, microglial cells, and L1-positive microglial cells. These microglial cells were co-cultured with cerebellar granule cells. For control, potential homophilic L1 interactions were blocked by the L1 antagonist antibody 324(AB 324). Only in the absence of this antibody, microglial cells showed increased neurite outgrowth compared to L1-negative microglial co-cultures (*p* < 005; Figure 3). These results indicate that L1 expressed by microglial cells stimulates neurite outgrowth via a homophilic L1 interaction.

### 2.4. L1-Positive Microglial Cells in the Adult Spinal Cord

**Figure 4:**
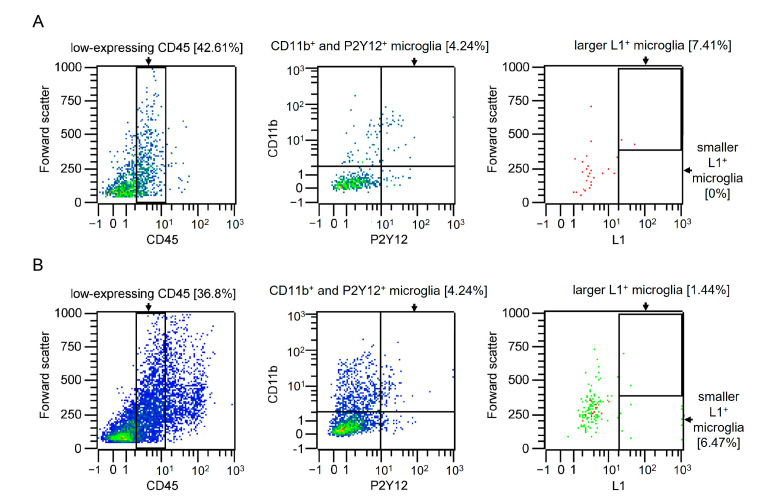
Subpopulations of L1-expressing monocytes in the adult healthy and injured spinal cords: (**A**,**B**) Flow cytometry of freshly isolated microglial cells from (**A**) non-injured (*n* = 1) and (**B**) injured pooled spinal cords of 10-week-old mice of both sexes (*n* = 1). Microglia-specific gates were set as described in Figure 1 (left and middle scatter plots). The right scatter plots show the size and the percentage of microglial cells that express L1. Color gradients in the scatter plots are representing the density of cells in different regions of the plot. Blue dots indicate a low cell number, green dots indicate a moderate cell number, and red dots indicate a high cell number.

Based on the observation that L1-positive microglia increase neurite outgrowth, we now ask whether there are microglial cells that express L1 in the healthy spinal cord and after injury (Figure 4). Cells were taken from 10-week-old healthy mice and mice 24 h after injury. Cells were stained for microglia using CD45, CD11b, and P2Y12 antibodies. In a healthy spinal cord, 7.41% of the isolated microglia were found to express L1 (Figure 4A) and forward scatter indicated that a subpopulation of L1-positive microglial cells displays a larger cell soma. In injured spinal cords, 7.91% of microglial cells express L1, 1.44% had a larger cell body, and 6.47% had a smaller cell body (Figure 4B).

### 2.5. Depletion of L1 from Monocytes Reduces Functional Recovery After Spinal Cord Injury

**Figure 5:**
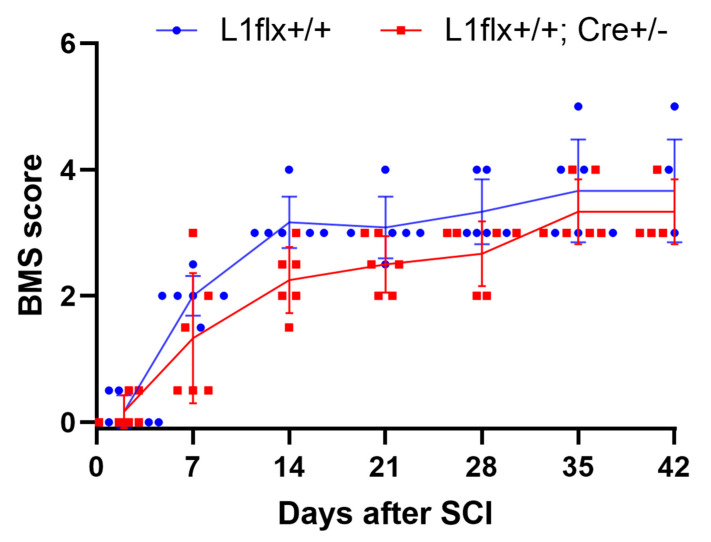
Mice with L1-depleted monocytes show reduced recovery after SCI. In the experimental group, homozygous L1 flox mice were crossed with heterozygous CX3CR1-creER mice L1 in which cre-recombinase expression was controlled by the monocyte-specific CX3CR1 promotor. After tamoxifen treatment, the experimental group contains L1-depleted monocytes. For control, homozygous L1 flox mice were treated with tamoxifen, indicating the presence of L1 in monocytes. The diagram shows the average BMS scores and individual data points of the two groups. Data are presented as mean ± SD (*n* = 6 mice/group). Statistical analysis was performed using repeated measures ANOVA.

Since L1 contributes to functional recovery after spinal cord injury [6,7,8,9,10], we asked whether L1-expressing monocytes contribute to this recovery. To analyze this, we crossed monocyte-specific CX3CR1-creER tamoxifen-inducible Cre-driver mice [23] with L1-flox mice [24]. In the experimental group, mice were heterozygous for CX3CR1-creER and homozygous for the L1 floxed LoxP site. After tamoxifen treatment, creER is transferred to the nucleus, and the L1 gene is abolished in monocytes. In the control group, these cells were only positive for the L1 floxed LoxP site but did not express cre recombinase. The function of the cre-recombinase system was tested by tamoxifen injection into 5-day-old L1flox or L1 CX3CR1creER mice. A decrease in L1 expressing microglia of CX3CR1creER mice indicated that the cre-driver system was functional (Figure S3). No difference in body weight was observed between the experimental group and control group up to 35 days after injury (Figure S4). In the L1-depleted experimental group, there was no difference in the Basso Mouse Scale (BMS) scores compared to the control group that was not subjected to cre activation (Figure 5).

### 2.6. L1-Expressing Monocytes Do Not Affect Lesion Volume or Myelin Damage

**Figure 6:**
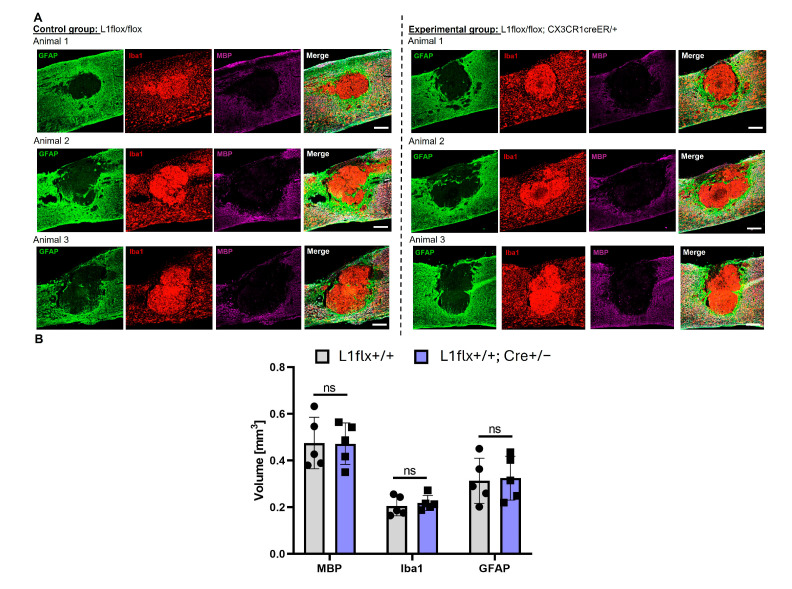
The lesion volume does not differ between the experimental groups without L1-expressing monocytes and wild-type littermates: (**A**) Representative images from three different animals per group show immunostainings of sagittal spinal cord sections that contain the lesion in the center. The green channel represents GFAP staining for astrocytes, the red channel represents Iba1 staining for monocytes, and the purple channel represents MBP staining for myelin. (**B**) The bar diagram shows the average volume of GFAP negative staining in the lesion, Iba1-positive filling of the lesion, and lack of myelin (MBP staining) around the lesion and individual data points from all experimental groups (circle: L1+/+ mice, square: Cre+/− with L1+/+ mice). Data are presented as mean ± SD (*n* = 5 mice/group). Data were analyzed using Student’s *t*-test. *p* > 0.05; ns—not significant difference.

Immunofluorescence analysis was performed after SCI at the T11 level. Glial fibrillary acidic protein (GFAP, green), ionized calcium-binding adaptor molecule 1 (Iba1, red), and myelin basic protein (MBP, purple) were used as markers for astrocytes, microglia, and myelin, respectively. The merged image combines all markers to illustrate the spatial relationships between astrocytes, microglia, and myelin in proximity to the lesion site (Figure 6A). The staining of the lesion site and surrounding tissue was compared between the control group, L1 flox/flox, as well as the experimental group, L1flox/flox; CX3CR1creER/+. No significant difference was observed by immunohistochemistry around the lesion in the presence or absence of L1 expression in microglia. The volume of the lesion measured with GFAP staining showed no significant difference between the groups. Also, the volume of microglial cells and monocytes in the lesion that were identified by Iba1 staining were not different between groups. MBP staining and the volume of lost myelin were also not different between groups (Figure 6B). These results indicated that L1 expressed by microglia does not affect the lesion volume, monocyte core in the lesion, and myelin surrounding the lesion.

### 2.7. Depletion of L1 from Monocytes Decreases Fiber Sprouting Caudal to the Lesion Site

**Figure 7:**
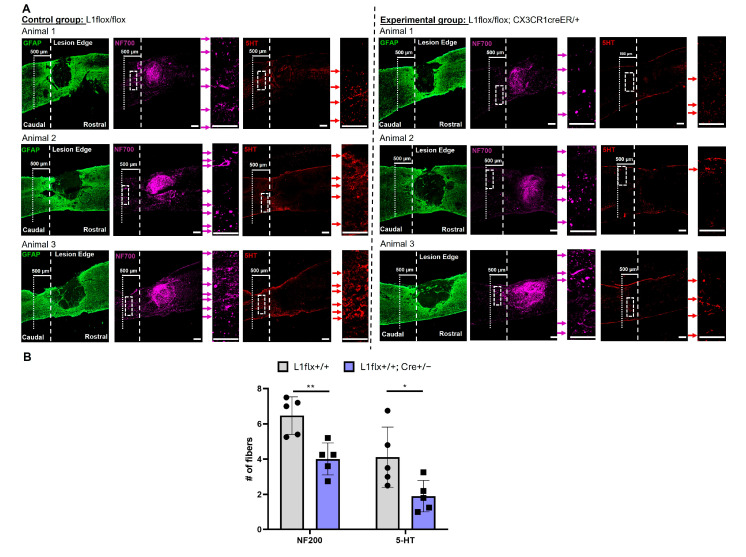
Increased numbers of NF200-immunoreactive and 5HT-immunoreactive fibers caudal to the lesion in wild-type mice compared to mice with L1-negative microglia: (**A**) Representative images show immunostainings in sagittal spinal cord sections with lesions in the center. The green channel shows the GFAP staining to identify the lesion, the purple channel represents NF200 staining, and the red channel represents 5-HT staining. The dotted lines on the right side indicate the caudal lesion edge. The dotted lines on the left side indicate the site where fibers were counted. The dotted box indicates the area that was zoomed in to show representative areas of fibers. The red arrows indicate 5HT-positive fibers, and the purple arrows indicate NF200-positive fibers. Scale bars = 200 µm. (**B**) The bar diagram shows the average numbers of NF200-immunoreactive and 5-HT-immunoreactive fibers and individual data points from all experimental groups (circle: L1+/+ mice, square: Cre+/− with L1+/+ mice). Data are presented as mean ± SD (*n* = 5 mice/group) and were analyzed using Student’s t-test. * *p* <0.05 and ** *p* < 0.01.

Since we observed a tendency of better functional recovery after SCI, when L1 was expressed in microglia, the sprouting of 5HT-immunoreactive fibers was determined caudal to the lesion site. We counted 5HT-immunoreactive and NF200-immunoreactive fibers up to 500 µm caudal to the lesion site (Figure 7A). The average number of 5HT-immunoreactive and NF200-immunoreactive fibers was higher in the control group with L1-expressing microglia than in the experimental group without L1-expressing microglia (Figure 7B).

## 3. Discussion

Unexpectedly, we found that a subpopulation of microglia expresses L1 in the normal brain. Hitherto, L1 was described to be expressed only by neurons in the central nervous system (CNS) and enteric neurons and Schwann cells in the peripheral nervous system. The identification of monocytes had been difficult because they were mostly only available when cultured after extraction from the CNS tissue. For a long time, it was difficult to discriminate between mononuclear phagocytes that had infiltrated the brain and activated resident microglia because of their similar composition of biochemical markers. In addition, the CNS environment triggers the differentiation of CNS-infiltrating macrophages to a neural-specific phenotype. This is only after the availability of single-cell sequencing to identify markers that differentiate microglia from CNS-infiltrating macrophages [22]. By using the microglia-specific marker P2Y12, we detected L1 expression in a subpopulation of microglia cells that was freshly isolated from the mouse CNS. Interestingly, the control microglia population in 7-day-old mice is more than 10 times higher than in 3-week-old mice. This change in L1 expression may reflect the change in the role that microglia perform during development versus the adult central nervous system. Transcriptome analysis performed with single-cell RNA sequencing from microglial cells at different ages indicated a considerable diversity in gene expression during early development and a decreased diversity in adult microglia [25,26].

During aging, a shift occurs in microglia from an anti-inflammatory to a pro-inflammatory state. Senescence and other age-dependent cell physiological changes may reduce the ability of microglia to sense changes in their environment [26]. During development, microglia play a role in myelinogenesis, oligodendrocyte progenitor cell differentiation, astrocyte maturation, synapse pruning, and circuit formation, as well as blood vessel development [27,28,29]. Likewise, L1 is a key player during development due to its receiving and transmitting of micro- and macro-environmental signals. On its own, L1 signaling triggers functions such as cell migration and neurite outgrowth [5]. Interestingly, cell migration was inhibited by an L1-function blocking antibody, and L1-positive microglia enhanced neurite outgrowth, which is reduced by the L1-function blocking antibody. Our findings suggested that L1-positive microglia guide neurite outgrowth during development. Furthermore, microglia play an important role in development [30,31] and determine neurite outgrowth in the retina. Expression of complement protein C1q by microglia limits retinal horizontal cell neurite outgrowth to the outer retinal synaptic layer [32]. In Drosophila, glial cells engulf fragmented axons in a microglial-like manner to facilitate larval development [33]. In addition, microglia regulate the outgrowth of dopaminergic axons in the developing mouse forebrain [34]. The immortalized mouse microglial cell line BV2 stimulates neuritogenesis of human neural progenitor cells following a pro-inflammatory stimulus [35]. Yet, microglia are not found in most embryonic brain regions comprising growing axons, suggesting that microglia may inhibit axon outgrowth [36]. Yet, microglia are essential for the differentiation of oligodendrocyte precursor cells into mature oligodendrocytes and the survival of oligodendrocyte precursor cells [37,38].

Spontaneous functional recovery after neurotrauma can indeed occur in the adult central nervous system [39]. It is believed that a partial recovery is mediated in part by neuroplasticity in the reorganization of neuronal circuitry and collateral sprouting of spared axons. Neonatal mice reached sham control levels of locomotion after spinal cord compression injury at the lower thoracic levels [40]. Regeneration of damaged axons through the lesion site is hindered in part by cell surface molecules on activated astrocytes [41]. In adult mice, astrocytes are activated and form a barrier around the lesion site to seal off this site from inflammatory signals. After a crush injury, neonatal mice exhibit a microglia-dependent and scar-free spinal cord repair [42]. Despite a low number of L1-positive microglia in the spinal cords of 10-week-old mice, mice tended to attenuate functional recovery after SCI if L1 was deleted from monocytes. In addition, mice with L1-positive microglia showed higher numbers of 5HT-immunoreactive and NF200-immunoreactive fibers rostral to the lesion site. Taken together with our finding that L1-positive microglia stimulate neurite outgrowth in vitro, we propose that microglia contribute to spontaneous recovery after SCI. L1 in the microenvironment of tumors displays anti-inflammatory properties [43]. However, we did not see any difference in the lesion volume or myelin loss caused by the contusion and subsequent inflammation in the injured spinal cords of mice with L1-positive microglia compared to mice that were depleted of L1-positive microglia. The low number of L1-expressing microglia might not be enough to attenuate the inflammatory response to SCI. Surprisingly, this low number of L1-positive microglia seemed to improve functional recovery by enhancing fibers rostral to the lesion site.

We would like to mention here that L1 also reduces the progression of AD in mouse models. L1 interacts with autophagy-related protein 12, which is essential for autophagy substrate selection for Aβ elimination [44]. Here, microglia come into play in neuroinflammation and clearance of Aβ aggregates [45]. In addition, an age-dependent decrease in the microglia-dependent phagocytosis of Aβ fibrils was observed [46], suggesting that L1-expressing microglia may clear Aβ aggregates and that the age-dependent decrease in the L1-expressing microglia affects the ability of microglia to clear Aβ fibrils. Thus, it would be interesting to study the role of L1-expressing microglia in AD animal models.

## 4. Materials and Methods

### 4.1. Animals

CB6F1/J mice were purchased from the Jackson Laboratory (Strain #:100007, Bar Harbor, ME, USA). L1-flox mice were obtained from Dr. Jean Hébert (Albert Einstein College of Medicine, Bronx, NY, USA) [24]. The CX3CR1-creER line was a kind gift of Dr. Longjun Wu (Mayo Clinic, Rochester, MN, USA) (Jackson Laboratory, strain #021160) [23]. Mice were maintained under standard and approved conditions at the facility of the Division of Life Sciences at the Nelson Biology Laboratories of Rutgers University. Both sexes were used in all experiments. For microglia culture, 0- to 2-day-old mice were used, and a cerebellar granule neuron culture from 5- to 7-day-old mice was used. For flow cytometry, 7- to 21-day-old and 10-week-old mice were used, and 10- to 14-week-old mice were taken for SCI. Genotyping was performed on tissue collected by ear clipping or tail biopsy. To confirm L1-flox and CX3CR1-creER genotypes, a polymerase chain reaction was performed. DNA was extracted with Bioline (Camarillo, CA, USA) MyTaq™ Extract-PCR Kits (cat# BIO-2112).

### 4.2. Antibodies and Reagents

Chemicals were obtained from Sigma-Aldrich (St. Louis, MO, USA) if not indicated otherwise. For cell culture, Hank’s Balanced Salt Solution (HBSS) (cat# 14175-095), Dulbecco’s Modified Eagle’s Medium, high glucose (DMEM) (cat# 11965092), B-27™ Supplement (50X), custom (cat# 0080085SA), and Penicillin–Streptomycin (5000 U/mL) (cat# 15070063) were from Thermo Fisher Scientific (Waltham, MA, USA). Horse serum (HS) (cat# 100-508, Lot# A24G) and fetal bovine serum (FBS) (cat# 900-108, Lot# E18H) were from Gemini Bio-Products (West Sacramento, CA, USA). Trypsin from bovine pancreas (cat# T4799), Collagenase Type IV (cat# C4-28), and DNase I from bovine pancreas (≥7500 BAEE units/mg solid) (cat# T9201). ViaStainTM (cat# CS2-0106) was from Nexcelom Bioscience LLC. (Lawrence, MA, USA). For flow cytometry, Myelin Removal Beads II (cat# 130-096-733), anti-mouse CD45 antibody coupled to VioBlue (cat# 130-110-802), REA Control Antibody for human IgG coupled to VioBlue (cat# 130-113-454), mouse CD171 (L1CAM) antibody coupled to APC (cat# 130-102-221), rat IgG 2a antibody coupled to APC (cat# 130-102-655), mouse CD11b antibody coupled to APC Vio770 (cat# 130-113-803), and recombinant human IgG1 coupled to APC Vio770 (cat# 130-113-435) were from Miltenyi (Gaithersburg, MD, USA). P2RY12 antibody coupled to PE (cat# 848004) and rat IgG2b isotype control coupled to PE (cat# 400635) were from BioLegend (San Diego, CA, USA). Anti-glial fibrillary acidic protein (GFAP) antibody, clone GA5 (cat# MAB360), anti-myelin basic protein (MBP) (cat# MAB386) antibody was from Millipore Sigma (Burlington, MA, USA), mouse anti-neurofilament 200 (NF200) monoclonal antibody was from MyBioSource (cat# MBS175078; San Diego, CA, USA), polyclonal goat serotonin (5-HT) antibody (cat# LS-C75755; Seattle, WA, USA) was from LSBio, and anti-ionized calcium binding adaptor molecule 1 (Iba1) antibody clone [EPR16588] was from Abcam (cat# ab178846; Waltham, MA, USA). Monoclonal antibody 557 that reacts with mouse L1 was prepared as described [47,48]. The L1 antagonistic rat anti-mouse L1 monoclonal antibody 324 was from Sigma-Aldrich (cat# CAS MAB5272). All secondary antibodies were from Jackson ImmunoResearch (West Grove, PA, USA).

### 4.3. Microglia Culture

Mixed glial cell cultures that contain predominantly microglia and astrocytes were from whole brains of neonatal CB6F1/J mice. Brains from males and females were pooled and incubated in 0.04% of trypsin, 4% of DNase I, and 0.8 mM of MgCl_2_ in HBSS for 15 min at 37 °C and mechanically dissociated by pipetting up and down. Glial cells were maintained in culturing flasks for 2 weeks in culture medium (DMEM + 10% horse serum + 10% fetal bovine serum + 1 × B27 + 50 U/mL penicillin–streptomycin). Every three days, the medium was renewed. After 2 weeks of culture, astrocytes had formed a monolayer, and microglial cells had expanded on this monolayer. Microglial cells were collected by shaking the culture flask for 2 h at 200 rpm and then separated from the astrocyte monolayer by tapping. Microglia cells were stained with ViaStainTM Acridine Orange and Propidium Iodide Staining and counted with a Cellometer Auto 2000 Cell Viability Counter (Revvity, Waltham, MA, USA).

### 4.4. Immunocytochemistry

Immunocytochemistry was performed essentially as described [10,49]. In brief, microglial cells were seeded onto poly-L-lysine-coated glass coverslips (500,000 cells per mL) and kept overnight in a tissue culture incubator. The next day, cells were fixed in 4% formaldehyde and then left at room temperature for 1 h in blocking solution (5% of bovine serum albumin and 0.3% of Triton X-100 in PBS). Primary antibodies were then added to blocking solution and incubated overnight at 4 °C. Microglia and monocyte markers were CD11b (1:100; Miltenyi) and Iba1 (1:1000; Abcam). L1 antibody 557 [47,48] was used at a 1:250 dilution. Cells were then washed thrice for 5 min at room temperature with PBS and incubated for 1 h at room temperature with secondary antibodies coupled to Alexa Fluor 488, Alexa Fluor 546, and Alexa Fluor 647. Finally, cells were washed thrice for 5 min at room temperature with PBS and mounted with ProLong™ Gold Antifade Mountant with DAPI (cat# P36931; Life Technologies, Carlsbad, CA, USA). Representative images were taken with an Axiovert200 Fluorescence Live Cell Imaging Workstation (Carl Zeiss, White Plains, NY, USA).

### 4.5. Flow Cytometry

Brains and spinal cords from CB6F1/J mice of both sexes were cleaned of meninges and blood vessels and cut into approximately 5 mm pieces. Tissue was incubated for 30 min at 37 °C with 1 mg/mL collagenase and 4% of DNase I in HBSS and then mechanically dissociated by pipetting up and down with a 1000 µL pipette. Myelin was removed by Myelin Removal Beads II (Miltenyi). Cells were stained with ViaStainTM Acridine Orange and Propidium Iodide Staining and counted with a Cellometer Auto 2000 Cell Viability Counter. After collection, cells were centrifuged at 300× *g* for 10 min at 4 °C, and the pellet was diluted in staining buffer (PBS + 2% FBS + 2 mM EDTA) up to 1 million cells per mL. The solution was split into two 100 µL portions. One portion was incubated for 10 min at 4 °C with fluorophore-conjugated antibodies against CD45, CD11b, P2Y12, and L1. The other portion was incubated with the corresponding secondary, control antibodies to estimate the background signal. Cells were washed thrice with 1 mL staining buffer and resuspended up to a volume of 100 µL. Dead cells and debris were excluded by staining with propidium iodide. Gates were set so as to eliminate unspecific backgrounds by incubation with control antibodies. Microglia express CD45 at a low level, whereas macrophages highly express CD45 [50]. Microglia and macrophages express CD11b, but only microglia express P2ry12 [51]. Thus, gates were set for low CD45 expression and then for CD11b and P2ry12 expression. Under these conditions, we analyzed the percentage of L1-expressing microglial cells.

### 4.6. Cell Migration Assay

In vitro, cell migration was assessed as described [10]. In brief, seeded microglial cells were maintained for 24 h until confluency. Scratching the monolayer with a 200 μL pipette tip generates a gap the size of which was immediately determined. After imaging, cells were either not treated (−) or treated with IgG from non-immune rats, the L1 agonist antibody 557, or the L1 antagonist antibody 324. After 24 h, images gaps were again taken to determine the gap closure percentage. For live cell imaging, a Zeiss Axiovert 200M inverted transmission-light microscope (Carl Zeiss, Jena, Germany) with a 10× objective, aperture of 0.25, and Axio Vision 4.6 software was used. The gap width was calculated using ImageJ software (Version 1.50b; NIH).

### 4.7. Cerebellar Granule Cells and Microglia Co-Culture

The preparation of cerebellar granule cells has been [10]. Briefly, cerebellar granule cells were allowed to settle for 1 h in an incubator. Microglial cells were prepared, as described in Section 4.3, and sorted using CD171 (L1CAM) magnetic beads (cat# 130-101-548, Milteny) to obtain L1+ and L1-microglia. One hour after seeding, cerebellar granule cells, L1+ or L1-microglial cells, were added (150,000/mL). For control, L1 antagonist antibody 324 was added. After 24 h, cells were fixed in 2.5% glutaraldehyde for 30 min at room temperature and stained with 1% toluidine blue and 0.1% methylene blue in 1% Na-tetraborate. Neurons were imaged using an Axio Observer.A1 microscope (Carl Zeiss) with a ×20 objective and AxioVision 4.6 software. The longest neurite lengths were measured from the edge of the cell body to the end of the process. Only neurites with a length equal to or greater than the diameter of the cell soma and only those without contact with other neurites or cell bodies were taken into account. ImageJ software was used for measurements.

### 4.8. Genotyping

For L1-flox genotyping, the L1-F (GAG CCA CCT GTC ATC ACG GAA C) and L1-R (CAT GGA TAA GAG GTT CTA GCA CTC) primers were used. For CX3CR1-creER genotyping, the Common-Cre (AAG ACT CAC GTG GAC CTG CT), WT-Cre-RV (CGG TTA TTC AAC TTG CAC CA), and Mutant-Cre-RV (AGG ATG TTG ACT TCC GAG TTG) primers were used. A thermal cycler (Mastercyler Ep-gradient, Eppendorf, Hamburg, Germany) was used for gene amplification: For L1-flox mice: 5 min at 94 °C, followed by 32 cycles of 15 s at 94 °C, 30 s at 60 °C, and 2 min at 72 °C. For CX3CR1-creER mice: 2 min at 94 °C, followed by 28 cycles of 15 s at 94 °C, 15 s at 60 °C, and 10 min at 72 °C. After the cycles, a 2 min elongation step at 72 °C was performed. After amplification, all samples were cooled to 4 °C.

### 4.9. SCI Mouse Model

For SCI experiments, L1flox mice were crossbred with microglia- and macrophage-specific CX3CR1 tamoxifen-inducible Cre-driver line. Mice homozygous for L1flox and heterozygous for CX3CR1creER (L1flox/flox; CX3CR1creER/+) were used. In the control group, mice were homozygous for only L1flox. Before surgery, mice were fed for two weeks by gavage with tamoxifen (500 mg/kg of animal/day [49]. After the two-week tamoxifen treatment, mice were allowed a one-week recovery. SCI was performed at spinal level T11 of 10- to 14-week-old male and female mice with the “Multicenter Animal Spinal Cord Injury Study” Impactor (Model A-3-8; Serial#: 04014) as described [49]. Shortly after surgery and on the first day thereafter, the mice received one 5 mL or 10 mL subcutaneous saline injection for females or males, respectively, followed by 25 mg/kg Cefazolin (cat# 054846; Covetrus, Portland, ME, USA). After surgery, mice were housed individually, and health and weight were monitored every day. The Basso Mouse Scale (BMS) [52] was used to monitor locomotion 1, 2, 3, 4, 5, and 6 weeks after surgery. A score of 0 represents no hind-limb movement, and scores increase with improved hind-limb joint coordination. A score of 9 represents normal walking.

### 4.10. Immunohistochemistry

Six weeks after injury, sagittal cryosections were prepared as described [49]. In brief, 1 cm of spinal cords containing the injury site in the center were post-fixed in 4% formaldehyde, and cryoprotected in PBS with increasing sucrose concentrations up to 20%. The tissue was frozen in Tissue-Tek^®^ O.C.T. Compound (Sakura, cat #45834; Torrance, CA, USA), and 20 μm thick serial sagittal sections were prepared. After antigen retrieval, sections were incubated with the following antibodies Iba-1, 1:1000; GFAP, 1:500, MBP 1:50; spinal fiber sprouting: 5-HT, 1:100; NF200, 1:500; GFAP, 1:500) followed by incubation with the corresponding Alexa Fluor 488-, Alexa Fluor 546-, and Alexa Fluor 647-conjugated donkey secondary antibodies (1:1000 in blocking solution) for 1 h at room temperature. Sections were then mounted with ProLong™ Gold Antifade Mountant with DAPI. Images were taken with an Axiovert200 Fluorescence Live Cell Imaging Workstation (Carl Zeiss).

### 4.11. Quantification of Immunofluorescence

For quantification of the lesion volume and fiber numbers, 20 μm thick serial sagittal sections throughout the entire spinal cord of all animals were analyzed. The sections were collected at a 100 μm distance.

For the determination of lesion volume, the lesion area was identified by the absence of GFAP staining, while demyelination was identified by the absence of MBP staining. Microglia and macrophages that had infiltrated the lesion were identified by Iba1 staining. Areas were measured with ImageJ software, and the volume was calculated according to the measured area and the thickness of the analyzed area.

For fiber measurements, fibers in an area up to 100 μm rostral to the lesion edge were counted in every section and averaged. The averages in every group were calculated from all animals.

### 4.12. Statistics

All experimental groups were blinded for data acquisition and analyses. Statistical differences between experimental groups were analyzed by student *t*-test, one-way ANOVA with Fisher’s protected least significant difference (PLSD) post-hoc test, or by repeated measures ANOVA using StatView Version 5.0.1 (SAS Institute Inc., New York, NY, USA), and Microsoft 365 Excel (Redmond, WA, USA). Graphs were prepared with GraphPad Prism 10 (GraphPad Software, Inc., Boston, MA, USA). Data are represented as Mean ± SD.

## 5. Conclusions

We here describe a subpopulation of microglial cells that expresses the cell adhesion molecule L1. This population stimulated neurite outgrowth in co-cultures with neurons. Since the number of L1-expressing microglial cells was found to decrease with age and since L1-positive microglial cells tended to improve recovery after spinal cord injury, we expect that L1-expressing microglia not only improve recovery after traumatic injury but may also attenuate the progression of AD and other neurodegenerative diseases.

## Data Availability

Data are available from the corresponding author upon reasonable request.

## Supplementary Materials

The following Supporting Information can be downloaded at: https://www.mdpi.com/article/10.3390/ijms26073285/s1.
