# Supplementary material for: Depletion of Cell Adhesion Molecule L1 from Microglia and Macrophages Reduces Recovery After Spinal Cord Injury
Source: Int J Mol Sci. 2025 Apr 1;26(7):3285. doi: 10.3390/ijms26073285 (PMC11989546; doi:10.3390/ijms26073285)
Supplement: Supplementary file 1 [file ijms-26-03285-s001.zip › ijms-3426760-supplementary.pdf]

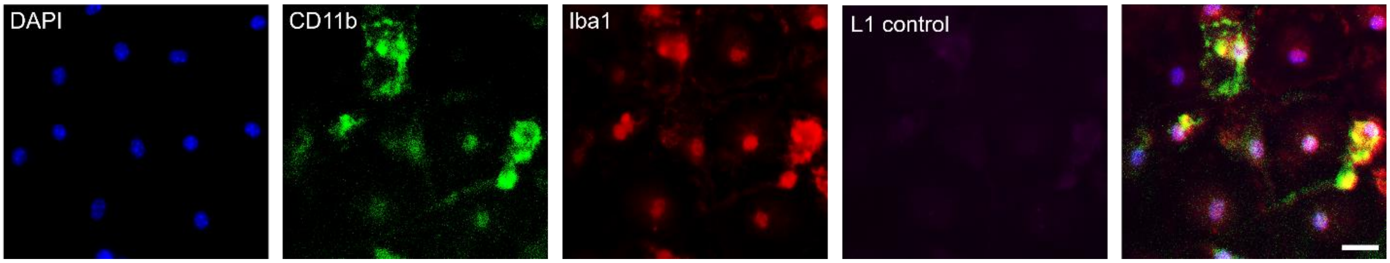

Supplementary Figure 1: Representative images of cultured microglial cells stained for the microglia markers CD11b (green) and Iba1 (red), and cell nuclei (DAPI, blue). As control only the secondary antibody (violet) was used as background control for the L1 staining. Scale bars = 30 µm.

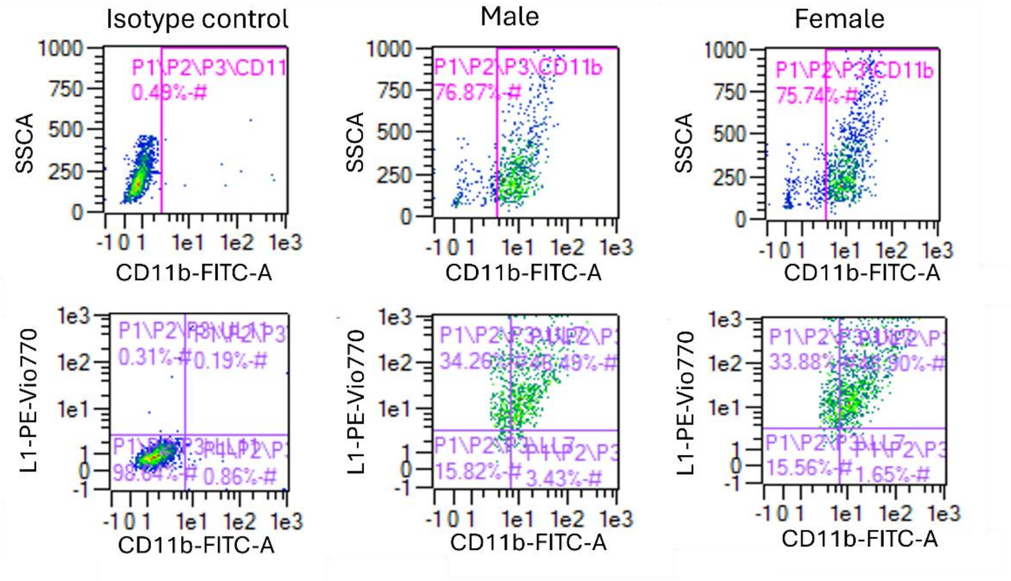

Supplementary Figure 2: Flow cytometry of freshly isolated microglia from pooled brains of 4-day-old mice (n = 3 of male and n = 3 of female). The gates were set to identify the microglia and monocytes. The isotype controls show Low background of the CD11b and L1 staining (left scatter plots). Comparison of male female mice did not show any differences of L1-expressing CD11b cells between the sexes (male = 46.49%; female = 48.9%).

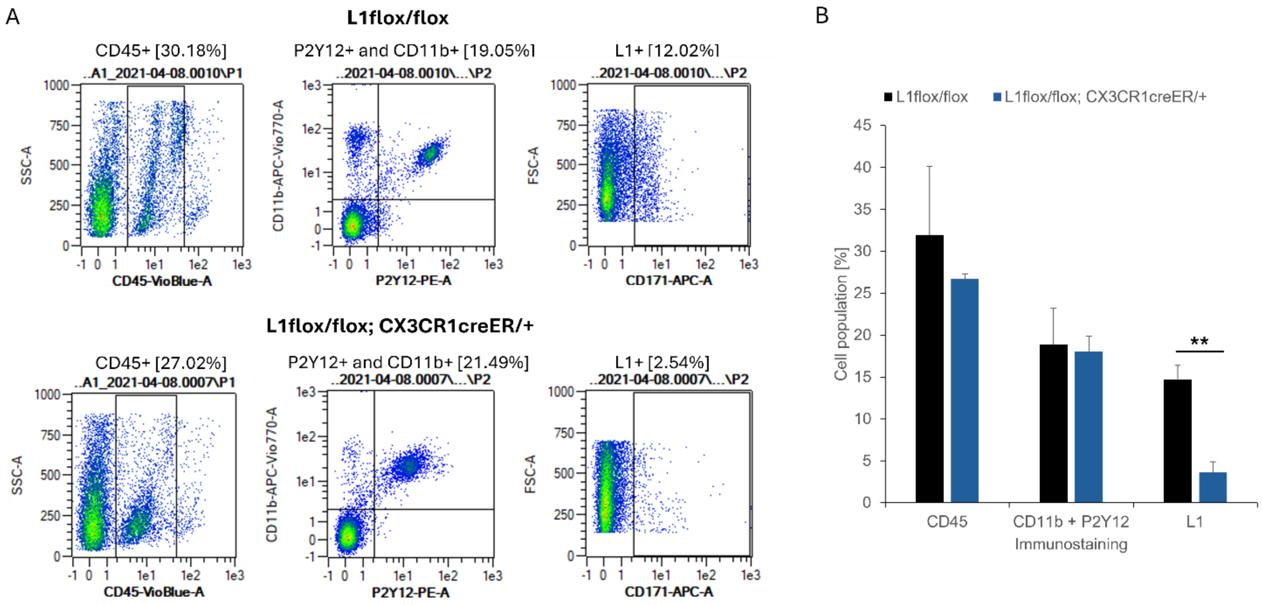

Supplementary Figure 3: Verification of the L1 knockout in microglia and monocytes of L1flox and CX3CR1creER mice. Three 5-day-old L1flox and CX3CR1creER mice and as control three 5-day-old L1flox mice were subcutaneously injected with 100 µg/mouse tamoxifen. Four days later, the mice were euthanized and cells extracted from the brains. Cells from individual animals were stained with CD45, P2Y12, and CD11b to identify the microglia population with flow cytometry and stained with L1 to analyze the percentage of L1-expressing microglia. (A) Representative scatter plots show flow cytometry analyzes of one animal from each group. (B) Bar diagrams show average percentage and SEM (n = 3 each group, 2 males and 1 female) of CD45, CD11b + P2Y12, and L1 stained microglia. The percentage of microglia that express L1 was significant lower in the L1flox and CX3CR1creER mice (student t-test, ** $p<0.01$). These results indicates that the Lox-Cre system is functional and depletes L1 from microglia.
